# Parturient Apoplexy1Read before the semi-annual meeting of the Pennsylvania State Veterinary Medical Association, Reading, October, 1896.

**Published:** 1896-12

**Authors:** J. Curtis Michener

**Affiliations:** Colmar, Pa.


### PARTURIENT APOPLEXY.[1]

By J. CURTIS MICHENER,
COLMAR, PA.

It is by request, and not of choice, that this subject is taken; and I open the discussion upon this mysterious disease for the purpose of acquiring, not imparting, information. Thirty years ago I could have given the cause, pathology, and treatment with the greatest of assurance, and felt confident of curing 90 per cent. of the cases, but more extended experience has re-

[1] Read before the semi-annual meeting of the Pennsylvania State Veterinary Medical Association, Reading, October, 1896.

moved conceit and demolished theories until there is very little of a positive character to build upon. Such being the case, it is becoming that the present paper should be confined to reports of cases and such practical points as have been acquired by experience, and leave the determining of the cause and pathological anatomy to the truly scientific investigator.

The literature of the subject abounds in numerous theories as to the cause and nature of the disease, and the widest range of treatment, all of which goes to show that it is very imperfectly understood. We meet statements by prominent authors which are conclusive, to my mind, that errors are made in diagnosis and irrational conclusions arrived at. For instance, Fleming quotes Franks as claiming that oxen and other domesticated animals are sometimes affected by it, and other authorities claim that it occurs even as late as the seventh week after calving, which is all nonsense. Cattle become stupid, paralyzed, and unable to rise by eating too much, especially when they have access to some concentrated feed, or that to which they are unaccustomed; and some cases that are unable to rise before, during, or soon after calving (and denominated parturient apoplexy) are simply cases of engorgement. Fleming says the attack is sometimes ushered in by a shivering fit. I do not believe that; but have seen them so nervous that they shook all over while leaning against the stall or pushing against the manger to keep from falling. Require them to step aside and they go down, and the shaking is over, and no rise of temperature follows, as when having a chill. It is a mistake to call the disease a fever. There is no rise of temperature except in the last stages with the lung complication. The peculiar and characteristic position, often met, of resting on the sternum and the head turned around toward the shoulders or flank (and will stay no other way), is not due "to contraction or tonic spasm of the cervical muscles of one side of the neck," but is a case of *hemiplegia*, and the fore- and hind-part come together because the muscles of one side retain their tone while those of the other side are paralyzed. The heat observed on the concave side arises from the parts being brought together, the same as a man is warm in the armpits.

I shall not attempt to criticise absurd theories of the cause and nature of the disease, but will state my opinion briefly, that you may crack it and see if it contains a kernel. No one can treat a disease rationally without knowledge of its nature and pathological conditions. This is essentially a congestive disease, characterized by loss of function. It attacks cows of middle or advanced age, which are "deep milkers." The part of the nervous system controlling the milk-secretion is highly developed, active, and very sensitive. This function has been at rest, and, a large part of the circulation going to nourish the fœtus, there occurs an easy birth; and now this predominant part of the nervous energy asserts itself with such force as to produce nervous shock, followed by brain-anæmia, resulting in loss of function; the digestive organs are torpid, the lacteal secretion is impaired or almost arrested, the uterus is contracted, throwing its former large blood-supply upon the great posterior aorta; the system is plethoric (nature has been providing for a great milk-supply)—now the animal lies prostrate, abnormal development of a part of the nervous system has deranged the working of the whole.

It makes an interesting case. The nervous equilibrium must be restored, the blood-circulation must be equalized, or death will soon follow. Shall we trust wholly to the recuperative powers of nature? Had it been done in every case I think more would have recovered. In this disease it is easy to do good or harm. There is room for the exercise of the greatest skill and care; what is good treatment in one case may be injurious in the next. I have settled down upon a few principles of practice, applied according to the circumstances:

1. Attention to position, to promote comfort and prevent injury. Secure several bags packed with straw to keep the cow upon the sternum and nose toward the ground. The position of the body and head will largely determine whether the case terminates in bronchial pneumonia and death after being on a fair way for recovery. Of course the same misfortune comes from giving large doses when the organs of deglutition are paralyzed.

2. Stimulate the circulation by outward applications: turpentine, mustard, hot-water packs, and warm clothing.

3. A stimulating purgative: sulphate of soda, one and one-half pounds; ginger, one ounce; nux vomica, two ounces; providing the patient can use the tongue and swallow well, and have the opportunity of giving it myself; otherwise she is better off without it. If there is much tympanitis puncture the rumen, using a large trocar; leave the canula in and cork, and introduce powdered charcoal at short intervals. Attend to evacuating the bladder and rectum; manually, if required. Place her nose in a pail of water occasionally, encouraging her to drink all she will.

4. Medication—caution! caution!! Best mix a little aconite and water, giving a small dose often to satisfy the owner. Sometimes, when stupor is great, pulse small, breathing not much disturbed and suspended for a moment at a time, large doses of diffusible stimulants seem to bring the answer; alcohol, aromatic spirits of ammonia, sulphuric ether, and sweet spirits of nitre in equal quantities, four ounces every two hours. This treatment is sometimes followed by a sudden collapse, and better results are obtained upon a whole by chloride of sodium, capsicum, and nux vomica, equal quantities by measure, one ounce every hour. These remedies are to be given with great caution and only when able to swallow. Am never able to say what will be the treatment in the next case; will be governed by the symptoms. May use opium or chloroform if the patient is wild and distressed, but will apply external stimulants and warm clothing every time.

Preventive treatment (or, rather, management) is of the most importance. Anyone owning an extra good cow, coming in profit for the third or more times, should be on his guard, not allowing her to become fat, living on poor pasture or hay the last month; but, above all, not changing the ration just before or after calving. Rub a little turpentine along the spine, and keep blanketed for three days. Some leading members of the Guernsey Cattle Club think they have found a preventive by letting her eat the afterbirth. No harm in that, or milking a few days before calving to encourage and establish the secretion.

Now, according to the request of our President, I will give a short account of cases treated since last spring. I treat all cases. In my early days of practice I knew which would get well and those that were not worth bothering with. Treat all cases until the lower jaw hangs pendulously, the breathing spasmodic and puffing, ears drooping, and nose cold and clammy. I shall not tire you with details.

Case I. *Feb.* 14. Jersey, six years of age; attacked eight hours after calving. Seen in four hours. Try new treatment: give vaginal injections of one ounce of creolin in two quarts of warm water every four hours. In twenty-four hours find improvement; continue injections and give one ounce each of spirits of nitre, ether, and ammonia every two hours. 15*th*.

Much better; continue injections only. 16*th.* Well as ever. All cases that I shall report had turpentine applied to the spine or the hot-pack, with warm, dry clothing.

CASE II. *March* 1. Eight years old; native. Attacked and seen on the second day. Gave purgative and the creolin injections, and gave the ether and ammonia treatment. Next day the cow was on her feet, making a rapid and complete recovery.

CASE III. *March* 8. Ten years old; native. Attacked twelve hours after calving. Same treatment as Case II. Grew rapidly worse, and died in thirty-six hours.

CASE IV. *March* 13. Grade Holstein, eight years of age. Attacked in fifteen hours. Creolin injections. 14*th.* No improvement; continue creolin and give the ether and ammonia every three hours. 15*th.* Better; continue same treatment. 16*th.* Improving; give purgative and continue creolin. 17*th.* Upon her feet, and purging. Made good recovery.

CASE V. *March* 18. Grade Holstein; only a little past five years old; third calf. Attacked in six hours; seen in twelve, a purge having been already given, and on tincture of aconite, twenty drops every hour, can still get up, but staggering; apply turpentine liniment, and continue same treatment. 19*th.* Much worse; stop aconite, and give the diffusible stimulant treatment. 20*th.* On her feet, purging and eating; gave aconite in very small doses. 21*st.* Grunting loudly and not eating much; pneumonia well established; apply hot-packs to the thorax; gave saltpetre, one ounce; tartar emetic, one drachm, three times daily; also one ounce sweet spirits of nitre every two hours. Died on the sixth day.

CASE VI. *April* 16. Twelve years old; native. Taken the tenth hour. Creolin injections and ether and ammonia treatment. 17*th.* Worse. Died on the third day.

CASE VII. *April* 25. Ten years; native. Taken in twenty-four hours. Same treatment as Case VI. 26*th.* Better. 27*th.* Worse; gave purgative. Died on the fourth day.

CASE VIII. *April* 3. Ten years; native. Taken in twenty-four hours. Same treatment. 4*th.* Better; continue the injections only. 5*th.* Better, eating, but cannot rise; gave one drachm of nux vomica three times daily. Up on the fifth day, and made a good recovery.

CASE IX. *May* 15. Nine years; Jersey. Taken in thirty-six hours. Same treatment. 16*th.* About the same. 17*th.* Better; continue injections only. 18*th.* Cow well.

Case X. *July* 6. Twelve years; native. Taken in six hours. Same treatment. Died on the second day.

Case XI. *July* 20. Nine years; grade Holstein. Taken in twelve hours. Same treatment; creolin and diffusible stimulant medicine. 21*st.* About the same; gave purgative. 22*d.* Purging, and better. 23*d.* Improving; use creolin injections only. 24*th.* Eating well; give one drachm of nux vomica three times a day; got up on seventh day, had bedsore, and lost one-quarter of the bag. Made good recovery, except being a "three teater."

Case XII. *July* 28. Ten years; native. Taken in thirty-six hours. Gave creolin injections and purgative. 29*th.* Better, and purging; gave three diffusible stimulant doses. 30*th.* Improving; used injections only. 31*st.* Well as ever.

Case XIII. Very old Jersey. Taken in eight hours. The owner's son, a doctor, came at bedtime. Their old favorite had calved that morning, and never appeared in better health and spirits. After dinner she was noticed to stagger, and refused to eat. In the evening she was found down, and taking no notice of anything. The doctor wished to know what I supposed ailed her, and I informed him she had what the farmers called milk fever. "But her bag is not inflamed," he replied. "I do not suppose it is," was my reply. "The name given by the farmers is a poor one." "What do you call it?" was the next query. "Parturient apoplexy." "But she does not have stertorous breathing, and was some four or five hours in losing her consciousness," was his reply. I informed him it was not true apoplexy, but that the name had been adopted by the veterinarians. "Well, what is it any way?" was his next query. I told him I was tired, and he should not bother me. "What are her chances? Expect she will die? You must come and see her anyway." Ordered tincture of aconite to be given in twenty-drop doses every hour, and creolin to inject the uterus every second hour; also a sheet to be wrung out of boiling water and spread over the body and well covered with dry blankets every second hour. I visited her at 8 A.M. the next morning, and the owner greeted me with a broad smile. "How is the old cow?" "She commenced to hold up her head and look brighter at 1 A.M., and just got on her feet as you were driving in." I found her a little unsteady in her movements, but looking for something to eat. Continued the same treatment every third hour, and on the third day found her as well as ever.

Case XIV. *August* 15. Eleven years old; native. Taken in thirty hours very bad in her head; tossing about violently at short intervals; breathing hurriedly and laboriously. Used creolin; gave purgative and one-drachm tincture veratrum viride every hour. Died on second day.

Case XV. *September* 20. Ten years old; native. Taken in twenty-four hours; found her upon her sternum, still noticing her calf. Gave her purgative; used her tongue well; put her on aconite every hour and used stimulating liniment. *21st.* Much worse. Raised the head and it would drop with a thud; flies would collect in the eyes and cause no annoyance; breathing slowly and quietly; respiration entirely suspended for a a minute at a time, occasionally. The owner and neighbors were astonished when I proposed going on treating the case, as they thought it was doctoring a dead cow. I told them that the days of miracles had not yet passed, and I had faith that the dead would come to life. Put her on alcohol, ammonia, and ether, and was rewarded the next day by seeing her with her head up, looking for something to eat. Continued her medicine in half-doses at intervals of three hours. *22d.* On her feet, well as ever.

Case XVI. *September* 24. Twelve years old; Jersey. Taken in thirty-six hours; very stupid; put her on ammonia and ether, and used the creolin injections. Grew steadily worse, and died on the fourth day.

Here we have seven deaths and nine recoveries. It was the first time I ever kept notes of cases, and I think some seasons would have given a better showing and some worse, the above being about the usual average. The creolin injections were a new departure, and I cannot say that they do any good. Post-mortems of four of the cases were made. Results negative; congestion of viscera, the lungs in particular; could not detect any lesion of brain or spinal cord; slight effusion into the arachnoid space in two of the cases.

---

Tincture of iodine in the drinking-water, alternated with compound tincture of cinchona-bark, will be found useful and effective in cases of diabetes insipidus occurring among green horses, following too severe work when convalescing from those infectious fevers incidental to transportation.

